# Formulated Palmitoylethanolamide Supplementation Improves Parameters of Cognitive Function and BDNF Levels in Young, Healthy Adults: A Randomised Cross-Over Trial

**DOI:** 10.3390/nu16040489

**Published:** 2024-02-08

**Authors:** Nadia Kim, Brenda Parolin, Derek Renshaw, Sanjoy K. Deb, Mohammed Gulrez Zariwala

**Affiliations:** 1 Centre for Nutraceuticals, University of Westminster, London W1W6 UW, UKsanjoy.deb@aru.ac.uk (S.K.D.); 2 Centre for Health and Life Sciences (CHLS), Coventry University, Coventry CV1 5FB, UK; ab9598@coventry.ac.uk; 3 Cambridge Centre for Sport and Exercise Sciences, Anglia Ruskin University, Cambridge CB1 1PT, UK

**Keywords:** palmitoylethanolamide, nutraceuticals, liposomal, cognitive function, students, university, BDNF, CANTAB, memory

## Abstract

Background: Palmitoylethanolamide (PEA) is an endocannabinoid-like lipid mediator which is naturally produced in the body and found in certain foods. The aim of this study was to assess the effect of a bioavailable formulated form of PEA (Levagen+^®^) on serum BDNF levels and parameters of cognitive function in healthy adults. Methods: A randomised double-blinded placebo-controlled cross-over trial was implemented to measure the effects of a 6-week 700 mg/day course of formulated PEA supplementation versus a placebo. Participants (n = 39) completed pre- and post-assessments of a lab-based cognitive test. Serum samples were collected to measure BDNF concentrations using an immunoassay. Results: A significant increase in serum BDNF levels was found following PEA supplementation compared with the placebo (*p* = 0. 0057, d = 0.62). The cognition test battery demonstrated improved memory with PEA supplementation through better first success (*p* = 0.142, d = 0.54) and fewer errors (*p* = 0.0287; d = −0.47) on the Paired Associates Learning test. Conclusion: This was the first study to report a direct beneficial effect of Levagen+^®^ PEA supplementation on memory improvement as well as corresponding increases in circulating neurotrophic marker levels. This suggests that formulated PEA holds promise as an innovative and practical intervention for cognitive health enhancement.

## 1. Introduction

Cognitive function encompasses a spectrum of mental processes such as attention, memory, executive function, and information processing speed, which collectively influence an individual’s ability to tackle complex tasks, solve problems, and adapt to novel situations [1]. Cognitive function is paramount for healthy young adults, particularly those in higher or university education. This cohort faces numerous intellectual challenges, including recalling key information for academic studies during examinations and assessments [2]. Furthermore, the onset of cognitive decline begins in early adulthood, with a recent study demonstrating a decline in cognitive and motor skills in complex tasks as early as 24 years of age [3]. This underscores the need to explore and implement cognitive enhancement strategies in young adults [4]. A recent review has identified 142 strategies for enhancing cognitive functions frequently employed by university students. However, several of these strategies are accompanied by intricate legal, social, and ethical considerations, rendering them subject to significant scrutiny [5]. Therefore, there is a need to develop safe and evidence-based methods to improve cognitive performance among this group while taking into account these complex factors. Safe and effective dietary interventions, such as adopting the Mediterranean diet [6] and supplementing with micronutrients such as B group vitamins, iron, and various polyphenols, represent evidence-based strategies to improve cognition [7,8,9,10].

While a balanced diet is essential for overall health, dietary supplements can offer higher concentrations of specific nutrients, antioxidants, or bioactive substances known to be beneficial for cognitive health [7]. Although complex and multifactorial, the mechanisms underlying the cognitive benefits of dietary supplements often intersect with neuroinflammation and brain-derived neurotrophic factor (BDNF) signalling. BDNF is a key protein required for maintaining normal neuronal function and is associated with improving memory, learning, and cognitive function [11]. Neuroinflammation, characterised by the activation of microglia and the release of pro-inflammatory cytokines in the brain, can detrimentally impact cognitive function [12]. For instance, polyphenols found in green tea and turmeric, as well as omega-3 fatty acids from fish oil, possess anti-inflammatory effects that can attenuate microglial activation and cytokine release, thus promoting a neuroprotective environment [13,14]. Neuroinflammation can impair BDNF expression and signalling, hindering synaptic plasticity and cognitive abilities [15]. Dietary compounds such as curcumin in turmeric and flavonoids in blueberries have been shown to upregulate BDNF expression [16,17,18]. They achieve this by activating signalling pathways such as the cAMP-response element-binding protein (CREB), which in turn enhances BDNF synthesis. BDNF is neurotrophic and promotes neuronal survival, synaptic plasticity, and neurogenesis, all contributing to improved cognitive function. In light of this emerging evidence, it is reasonable to propose that dietary supplements and nutraceuticals with anti-inflammatory properties, such as palmitoylethanolamide (PEA), have the potential to positively influence cognitive health by indirectly upregulating BDNF [19].

PEA is an endocannabinoid-like lipid mediator which is naturally produced in the body and found in foods such as egg yolk and peanut oil [19,20]. PEA supplementation has many health benefits, particularly in relation to inflammation and pain [21]. As PEA has been shown to cross the blood–brain barrier in rats [22], it was thought that it could also impact certain neural pathways, with both direct and indirect pathways identified. The primary receptor for PEA is peroxisome proliferator-activated receptor alpha (PPAR-α), whose activation is suggested to be responsible for PEA’s anti-inflammatory action [23]. Additionally, PEA has been reported to be involved in the autacoid local inflammation antagonism mechanism, downregulating the degranulation of mast cells [24]. The indirect effects of PEA include inhibiting the enzyme fatty acid amide hydrolase, which contributes to the degradation of cannabinoids [25]. Increased availability of PEA, therefore, increases the concentration of cannabinoids, which subsequently modulate factors of stress [26], neuroinflammation [27], and cognition [28,29].

The majority of previous studies involving PEA have focused on in vitro models or on animals or humans with health conditions, such as neurological disorders or inflammation, or on different sources of chronic pain through cannabinoid receptors [30,31,32,33]. In a recent study employing a murine model, an improvement in depressive symptoms was observed upon PEA administration [34], and in a cellular model, PEA has been suggested to exert neuroprotective effects through the modulation of microglial cells and proinflammatory molecules [34]. Beneficial effects of PEA supplementation were reported on expressive language and cognition in two case reports of autistic children [35]. A clinical trial involving stroke patients with mild cognitive impairment found that co-supplementation with PEA (1400 mg/day) in combination with luteolin (a flavonoid supplement commonly found in fruit and vegetables) for 60 days improved cognitive function [36]. The combined PEA/luteolin clinical investigation showed promising outcomes but did not demonstrate the application of PEA supplementation alone or offer insight into its application to the general population. Despite these promising outcomes, few studies have examined the relationship between PEA supplementation and cognition in a healthy general population. Such studies may reveal promising insights for the potential application of PEA supplementation in the context of cognitive health, an area of great significance to society. Therefore, this study aimed to examine, for the first time, the effect of PEA supplementation on markers of cognitive function in a healthy population currently enrolled in a higher education or university degree course.

## 2. Materials and Methods

### 2.1. Study Design

A randomised, double-blinded, placebo-controlled cross-over design was employed to investigate the effects of PEA supplementation on cognitive health in university students. This design allowed for minimised selection and allocation bias and rigorous control of potential confounding factors and ensured that each participant received both the PEA supplementation and a placebo in a counterbalanced order, with a washout period in between to minimise carryover effects [37,38]. The study involved a longitudinal design with a total of four scheduled visits, distributed across the duration of two academic semesters to comprehensively capture the potential impact of academic-related factors on cognitive function [39]. During each on-site visit, dynamic changes in cognitive function and BDNF levels that may occur over the course of an academic year were measured. The follow-up cognitive assessments were scheduled to coincide with periods of increased academic assessment, such as mid-term and final end-of-the-year examinations and assessments, a period where improved cognitive function may be of additional benefit and where participants may experience increased periods of stress [40].

### 2.2. Participants

The study included healthy male and female full-time university students from London universities aged 18 years and above who were fully enrolled in their course and who continued their course until the end of the intervention. Healthy participants were selected for this study to mitigate the confounding effects of long-term health conditions, such as high BMI or blood pressure. Such conditions are associated with chronic low-grade inflammation, and given that PEA is suggested to act via anti-inflammatory pathways [41], it was thought that this may have otherwise confounded the outcomes of the study. The following exclusion criteria were applied: those with any chronic health condition, neurological disorder, or learning disability; smokers; those consuming more than 14 units of alcohol per week; those on medication; those taking dietary supplements, including herbal remedies; and those who were concurrently volunteering for other research studies. Additional exclusion criteria included obesity; being pregnant; breastfeeding or trying to conceive; and people undergoing or planning any medical, dental, or orthodontic procedure. An online, open, cross-sectional survey targeting university students was locally disseminated through social media platforms such as LinkedIn and Instagram, utilising a purposive snowball sampling technique [42]. To screen for the criteria, participants’ survey responses were collected anonymously using the survey platform JISC (jisc.ac.uk, Bristol, England). Body mass index (BMI) and blood pressure were measured during the first on-site visit. Only those with a BMI between 20 and 30 and blood pressure below 140/90 mmHg were eligible for the study.

The study was explained in detail to each eligible participant by a research team member during the initial interview. This involved providing a copy of the Participant Information Sheet and outlining the study aims, procedures, and potential risks and benefits. At the end of the explanation, a signed consent form was obtained from each participant to indicate their willingness to participate in the study. The study was approved by the School of Life Sciences Ethics Committee, University of Westminster, in accordance with the ethical standards of the Helsinki Declaration of 1975 (Application ID: ETH2122–1031). The clinical trial was registered with ClinicalTrials.gov (Identifier: NCT06225440). Data were collected over teaching semester periods from September 2022 to May 2023 in a university laboratory in London.

### 2.3. Intervention

Participants willingly enrolled in the study, which comprised two distinct trial arms, with the order of assignment determined by a randomly generated sequence using GraphPad Prism (Version 9.4, Boston, MA, USA). Initially, participants were designated to either the PEA or placebo supplementation intervention. A six-week washout period was incorporated before commencing the alternative trial arm.

In the PEA group, participants were administered two capsules of PEA in a formulated form known as Levagen+^®^ (Gencor Pacific Limited, Lantau Island, Hong Kong). Each capsule is 350 mg, containing 300 mg PEA and 50 mg formulation excipients resulting in a total daily dosage of 600 mg PEA. Levagen+^®^ comprises PEA formulated with a proprietary delivery system (LipiSperse^®^, Pharmako Biotechnologies Pty Ltd., Sydney, Australia) that reduces the hydrophobic nature of PEA and has been shown to increase PEA bioavailability significantly as compared with its standard form [43]. Conversely, the placebo group received capsules containing equivalent amounts of microcrystalline cellulose without any active ingredients. Participants in both groups were directed to take two capsules simultaneously daily for a duration of six weeks. Capsules for both arms were sourced from Power Health Products Ltd. in York, UK, maintaining uniformity in appearance with identical size, colour, and shape so participants and researchers could not distinguish between treatments. Dosage and supplementation length were chosen based on the safety and efficacy reported in previous studies investigating aspects of physical and mental health [44,45,46].

### 2.4. Blood Sampling and Sample Handling

Phlebotomy was performed systematically to ensure that all participants received appropriate care and comfort. Trained phlebotomists took a single blood sample from each participant during each on-site visit, using a blood collection adaptor Luer Adapter (23 G Vacuette, Nipro, Osaka, Japan) and Serum Separator (8.5 mL) vacutainer by BD (Becton Dickinson, Eysins, Switzerland). The serum tube was left at room temperature for 45 min to coagulate, followed by a 15 min centrifugation at 710× *g* and 4 °C (Hettich 340 r, Hettich GmbH & Co. KG, Tuttlingen, Germany). The supernatant was immediately aliquoted (TubeOne^®^ microtubes, Star Lab, Milton Keynes, UK) and stored at −80 °C to maintain stability and integrity until further analysis was conducted [47]. The sample preparation was in line with BDNF assay manufacturer guidelines for sample preparation.

### 2.5. BDNF Analysis

BDNF analysis was conducted utilising the Biosensis CE Marked BDNF Rapid enzyme-linked immunosorbent assay (ELISA) Kit (Cat#: BEK-2211-1P-CE, Biosensis Pty Ltd. Thebarton, Australia), chosen for its established sensitivity, reliability, and suitability for the intended purpose of assessing BDNF levels. Specifically, the intra-assay coefficients of variance (CVs) were as minimal as 1%, underscoring the precision within a single assay run, while inter-assay CVs reached merely 5% (*p* = 0.392), confirming the consistency and reliability of measurements across different assay runs [48]. This kit comprises a pre-coated monoclonal anti-BDNF capture antibody, a biotinylated anti-BDNF detection antibody, and horseradish peroxidase (HRP)-conjugated streptavidin. Upon adding a substrate (3,3′,5,5′-tetramethylbenzidine, TMB), a colour reaction product is generated, directly correlating with the concentration of BDNF present in both sample specimens and protein standards.

Standard solutions of known BDNF concentrations provided in the kit were prepared to construct a standard curve, enabling the quantification of BDNF levels in test samples. Test serum samples and controls were added to the ELISA plate wells, where BDNF was present in the samples bound to the immobilised anti-BDNF antibodies on the plate. BDNF was detected using a biotinylated anti-BDNF antibody and subsequent enzymatic reactions. The absorbance of the samples was measured at 450 nm wavelength using a plate reader (POLARstar Omega, BMG Labtech, Ortenberg, Germany). BDNF concentrations in the test samples were determined by comparing the absorbance values to the standard curve generated from the known BDNF concentrations using regression analysis GraphPad Prism Version 9.4.

### 2.6. Cognitive Function Assessment

Cognitive performance was assessed using the Cambridge Neuropsychological Test Automated Battery (CANTAB©), a battery of choice—Core Cognition (©2022 Cambridge Cognition Ltd. version 1.7, Cambridge, UK). CANTAB^®^ software (Cambridge Cognition Ltd. version 1.7, Cambridge, UK)provides a rigorous and objective means of evaluating cognitive abilities, offering insights into specific cognitive domains [49]. The Core Cognition battery was explicitly utilised to measure various cognitive domains, including attention, working memory, spatial memory, executive function, and verbal memory. This battery is a well-validated and widely used tool for evaluating cognitive function in healthy individuals and individuals with neurocognitive disorders [50]. Furthermore, this battery has been used in previous studies to assess the effect of dietary supplements on cognitive function [51,52].

A touchscreen tablet (Apple iPad 9.7, 2017) loaded with the Cambridge Cognition CANTAB^®^ System application was provided to the participants. The assessments took place in a private and quiet room with minimal distractions. The room was designed to create a comfortable testing environment, with appropriate lighting and a thermoneutral temperature. The tests were designed to last 21 min. Details of each test and the outcome variables are outlined in Table 1.

### 2.7. Statistical Analysis

As initial assumptions and tests of normality were met, a paired *t*-test was used to compare the change score of all outcome variables between baseline and follow-up for the placebo and PEA conditions. These variables included serum BDNF and cognitive function outcomes of the Paired Associates Learning (PAL) recommended standard (PALFAMS and PALTEA), the Rapid Visual Information Processing (RVP)—3 targets (RVPA, RVPMDL, and RVPPFA), and the Spatial Working Memory (SWM) recommended standard (SWMBE and SWMS) tests. Furthermore, correlational analysis was performed to assess the relationship between changes in BDNF and cognitive function outcomes following PEA supplementation. A priori sample size calculations were determined with BDNF as our primary outcome, with an expected effect size of 0.5 based on data from previous research [46,47]. To achieve this effect with a statistical power of 80% to detect significant differences at an alpha of 0.05, a sample size of 34 was required. Effect sizes are reported as Cohens D effect, interpreted as 0.3 = small effect, 0.5 = moderate effect, and 0.8 = large effect. The statistical software package of G*power was used to determine sample sizes, while all other analysis was conducted on SPSS (IBM, Chicago, IL, USA) and Microsoft Excel (Microsoft, Redmond, WA, USA).

## 3. Results

### 3.1. Demographics

**Figure 1:**
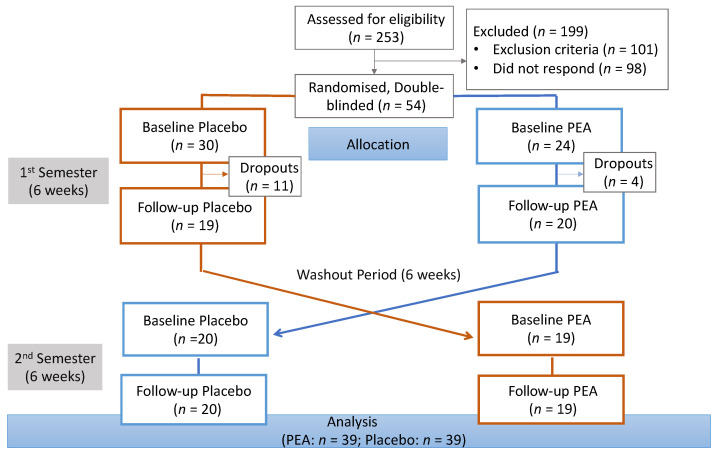
Overview of the recruitment process and random allocation of participants to the PEA and placebo groups.

A total of 54 participants were recruited and completed the first experiment, however, 15 participants dropped out subsequently (27% dropout rate), leaving 39 participants retained throughout the course of the study. Figure 1 provides an overview of the recruitment process and random allocation of participants in each group. Of these 39 participants (mean ± SD age: 22 ± 4.68 years; BMI: 22.05 ± 0.4), 64% were female (N = 25), and the remaining 36% were male (N = 14). The participants represented a diverse ethnic mix (White Caucasian: 43%; Black: 26%; South Asian: 26%; and other: 5%).

### 3.2. BDNF Measurement

**Figure 2:**
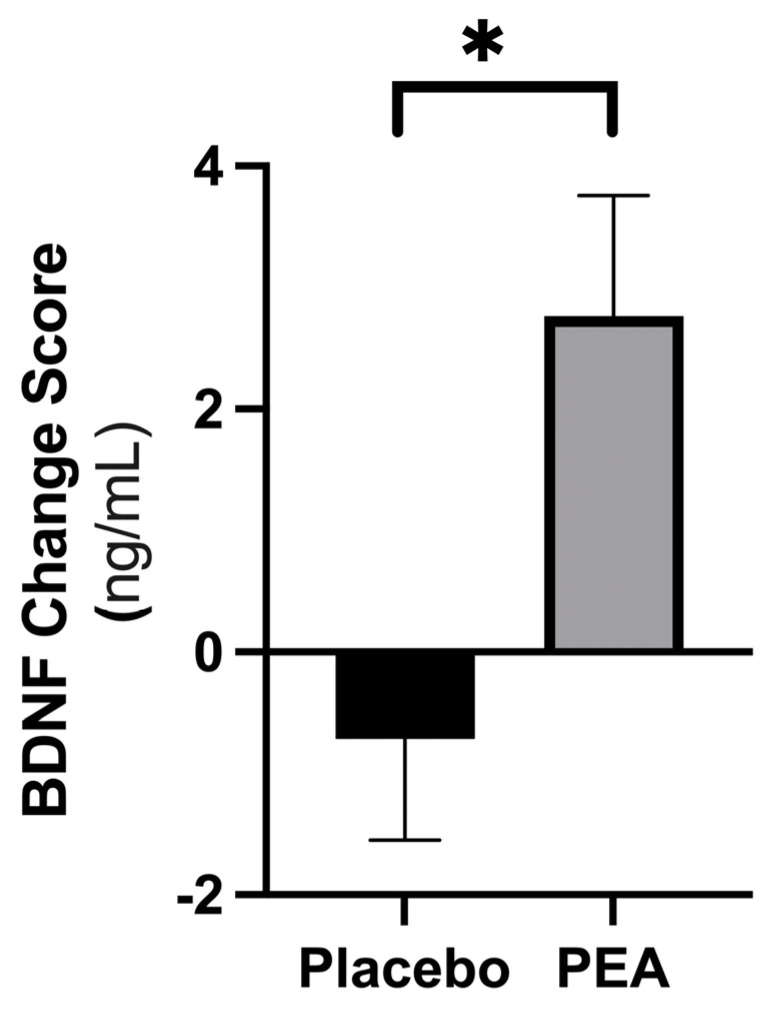
The change score of Levagen+^®^ PEA supplementation placebo from baseline to follow up on serum BDNF levels as compared with the placebo over the supplementation period. Data are expressed as mean ± SEM. * Denotes significant difference (*p* < 0.05).

Following six weeks of supplementation with Levagen+^®^ PEA, the expression of serum BDNF significantly increased by 2.76 ± 6.12 ng/mL (*p* = 0.0005) compared with no change in the placebo (−0.72 ± 7.16 ng/mL; *p* = 0.39). This difference was significantly different (*p* = 0.0057, d = 0.62), demonstrating that PEA supplementation had a significant moderate positive effect on BDNF (Figure 2). When investigating the order effect, no difference was found between phase 1 and phase 2 of the data collection periods (*p* > 0.05), suggesting the improvement in BDNF could be attributed to PEA supplementation alone. Correlation analysis did not find a significant relationship between change in BDNF and change in PAL First Attempt Memory Score (PALFAM—representing the number of correct boxes remembered on the first attempt; Table 1) (r = 0.21; *p* > 0.05).

### 3.3. CANTAB^®^ Cognition Function Assessments

**Figure 3:**
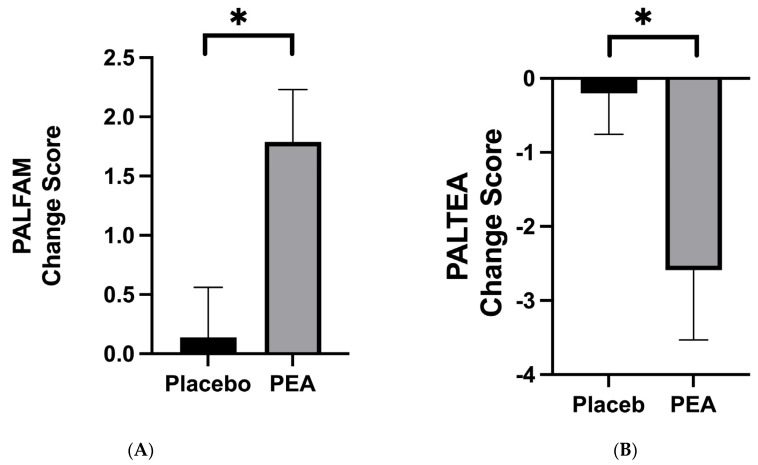
The change score of Levagen+^®^ PEA supplementation compared with the placebo from baseline to follow-up on memory, as determined by the CANTAB^®^ cognitive function test. (**A**) represents better success (Paired Associates Learning First Attempt Memory Score; PALFAMS) and (**B**) represents fewer errors during the memory test (Paired Associates Learning Total Errors Adjusted; PALTEA) following supplementation. Data are expressed as mean ± SEM. * Denotes significant differences (*p* < 0.05).

Overall, there were significant improvements in outcomes related to memory with Levagen+^®^ PEA supplementation. The PALFAMS was found to increase from baseline (15.09 ± 3.67) to follow-up (16.79 ± 2.97) by 1.71 ± 2.8 (*p* = 0.0005) with PEA supplementation. No change was reported in the placebo group (baseline: 16.15 ± 3.02 and follow-up: 16.40 ± 3.22; *p* = 0.5672), suggesting that PEA supplementation evoked a significant improvement in PALFAMS compared with the placebo (*p* = 0.0142; Figure 3A) with a moderate effect size (d = 0.54). Similarly, PALTEA was improved with PEA supplementation (baseline: 7.90 ± 10.37 and follow-up: 5.39 ± 7.60; *p* = 0.0129), but not with the placebo (baseline: 5.79 ± 6.74 and follow-up: 5.59 ± 7.89; *p* = 0.72). Accordingly, a significant improvement was observed when comparing the change score between PEA and the placebo (*p* = 0.0287; d = −0.47 Figure 3B). No trial order effect was observed with PALFAMS or PALTEA.

There were no supplemental effects on outcomes related to attention or psychomotor speed from PEA compared to the placebo in either RVPA (*p* = 0.541; d = 0.09), RVPMDL (*p* = 0.874; d = 0.01), or RVPPFA (*p* = 0.862; d < 0.001). Equally, outcomes of executive function also did not change with PEA supplementation (SWMBE: *p* = 0.8761; d = 0.04 and SWMS: *p* = 0.509; d = 0.15).

## 4. Discussion

This study is the first to report that supplementation with formulated PEA significantly enhanced circulating serum BDNF and improved memory in a healthy adult population. These findings also allude to a correlation between BDNF and memory, suggesting PEA may potentially mediate its cognitive-enhancing properties by increasing BDNF levels. Several pre-clinical studies have demonstrated PEA can influence neurobehavioral functions through oxidative and inflammatory mechanisms [17]. This may contribute to neuroplasticity through increased neural viability, survival, and BDNF upregulation [48], which may subsequently explain the improvement in memory observed. These findings lay the foundations for formulated PEA (Levagen+^®^) to be considered an effective nootropic supplement that can be used by populations to support academic study and by the wider general public to improve cognition.

This study observed a significant moderate increase in serum BDNF levels in the PEA treatment group compared with the placebo, which builds on pre-clinical research in animal models that also demonstrated an enhanced BDNF expression [49,50]. Moreover, our observations support prior research examining the effects of various anti-inflammatory nutraceuticals on BDNF levels. Investigations into anti-inflammatory compounds such as curcumin [51], zinc [52], probiotics [53], polyphenols [54], and carotenoids [55] have provided valuable insights into the potential influence of diverse dietary interventions on BDNF. A systematic review of 48 recent human interventions has reported mixed outcomes on BDNF concentrations for the various dietary interventions [56]. For example, a study employing a similar design that investigated the effect of whole coffee cherry extract (WCCE), a supplement rich in polyphenols, observed a similar increase in BDNF levels compared with the control (*p* = 0.04, d = 0.71) [36]. Comparatively, some studies on zinc supplementation have reported a significant enhancement in circulating BDNF levels (SMD: 0.31, 95% CI: 0.22–0.61) [52]. Supplementation with macular xanthophylls, a group of carotenoids, was associated with a significant increase in blood serum BDNF levels and a concurrent enhancement in cognitive performance within a healthy study cohort [18]. The results from the current study complement the existing evidence and provide further insights into the potential mechanistic relationship between PEA and BDNF in humans.

Overall, these neural changes would contribute to neural cell development and homeostasis [53], which in turn could impact mental health and cognition. In fact, BDNF is especially abundant in memory-related structures, such as the hippocampus and amygdala [57]. In addition, in some mental disorders where cognition is affected, BDNF is low [58]. Our study suggests there may be a trend that correlates enhanced memory and elevated serum BDNF levels within the PEA treatment group. Indeed, various nutraceuticals, such as curcumin, blueberries, and red grape, have shown a significant relationship between BDNF and cognition [59,60]. For example, a study on dietary supplementation with aloe polymannose multi-nutrient complex has demonstrated a correlation between elevated BDNF levels and improved cognition score (r = −0.53, *p* = 0.04) [54]. Another intervention showed that flaxseed oil supplementation had significantly positive effects on memory and BDNF concentration (*p* < 0.05) [61]. Additionally, mental training has been shown to increase both BDNF and memory [15], while low BDNF correlated with cognitive impairment [15]. As PEA and BDNF have been studied in preclinical studies or populations with health conditions [17], the current study adds promising evidence of the impact of a formulated PEA in supporting cognition in a healthy population.

Our results demonstrate that PEA supplementation was associated with improved performance in one of the domains of cognitive skills, specifically memory. CANTAB^®^ scores revealed a significant improvement in memory recall (PALFAMS) and a significant decrease in the total error rate (PALTEA) during pattern recall from baseline to endpoint in the PEA group, while no such changes were observed in the placebo group. In prior randomised trials involving different nutraceuticals, including docosahexaenoic acid [62] and polyphenols sourced from grapes and blueberries [63], significant enhancements in memory assessed by CANTAB^®^ have been documented. Noteworthy, not all results obtained from the CANTAB test battery in our study reported any effects with PEA. The Paired Associates Learning (PAL) test, a component of CANTAB^®^, is employed to detect memory-related issues [64]. PAL is considered one of the most sensitive means of measuring memory and learning ability and has been used for more than 100 years in human neuropsychopharmacological studies, and the CANTAB^®^ PAL has been in use for 30+ years in a multitude of studies [64].This study suggests that PEA may contribute, in particular, and perhaps preferentially, to an improved capacity for learning, knowledge retention, and recall.

It is important to highlight the limitations of the study design. While we observed a dropout rate of 27%, this is not uncommon in clinical trials and cross-over designs, as reports suggest typical dropouts of between 5 and 25% [65]. While it was not possible to ascertain the specific reasons for dropouts, they may have been influenced by extensive supplementation and a washout period of a minimum of six weeks, which was implemented to minimise any treatment carryover effects [66]. Furthermore, a priori sample size calculations suggested 54 participants were required to observe a change in BDNF at a statistical power of 95%. While the dropout rates meant we did not achieve this, if the minimum recommended statistical power of 80% was adopted [67], then 34 participants were required. Therefore, the final sample size for this study was appropriate to detect statistically significant changes. We also did not report an order effect; therefore, the dropouts in the first phase of data collection did not unduly affect the main outcomes. A lack of absolute control over dietary intake throughout the intervention is another potential limitation of this study. However, it is important to consider that the nature of the intervention itself may mitigate this. PEA, the focus of this study, is not commonly found in substantial quantities within typical dietary sources [68]. For instance, the daily recommended portion of soybeans, which contain the highest known concentration of PEA among food sources, provides only about 5.4 mg of PEA [69]. This amount is considerably lower than the 700 mg/day dosage administered through the supplement and may be deemed ineffectual to exert any confounding effects.

## 5. Conclusions

Although these findings provide a promising avenue for further research and application in cognitive function for PEA in general, it is pertinent to point out that the form of PEA used in this study, Levagen+^®^, was a formulated form that has been shown to be significantly more bioavailable than native PEA and may therefore have evoked more sustained and potent effects resulting in the beneficial outcomes observed. In conclusion, our study has provided valuable insights into PEA supplementation’s cognitive and neurotrophic effects in a cohort of healthy young adults. The increase in serum BDNF levels within the PEA treatment group, accompanied by enhancements in memory and cognitive performance, suggests the potential of formulated, bioavailable PEA supplements to be a practical and safe nootropic nutraceutical.

## Figures and Tables

**Table 1:** Parameters of the CANTAB^®^ cognition test battery.

Endpoint Tested	Attention and Psychomotor Speed	Memory	Executive Function
Test	Rapid Visual Information Processing (RVP)	Paired Associates Learning (PAL)	Spatial Working Memory (SWM)
Outcome Measure	RVPA: The sensitivity to the target regardless of response tendency. RVPMDL: The median response latency on trials where the subject responded correctly. Calculated across all assessed trials. RVPFA: The number of sequence presentations that were false alarms divided by the number of sequence presentations that were false alarms plus the number of sequence presentations that were correct rejections: (false alarms ÷ (false alarms + correct rejections)).	PALFAMS: The frequency with which participants chose the correct box on their first attempt. PALTEA: The number of trials required to locate the pattern(s) correctly and the memory scores and stages completed.	SWMS: The possibility of the participant using a certain searching strategy. SWMBE: The number of times an individual incorrectly revisited an emptied box.
Task Format	A white box was shown in the centre of the screen, inside which digits from 2 to 9 appeared in a pseudo-random order, at the rate of 100 digits per minute. Participants were requested to detect target sequences of digits (for example, 2-4-6, 3-5-7, 4-6-8). When the participant saw the target sequence, they had to respond by selecting the button in the centre of the screen as quickly as possible. The level of difficulty varied with either one or three target sequences that the participant had to watch for at the same time.	Boxes were displayed on the screen and were “opened” in a randomised order. One or more of them contained a pattern. The patterns were then displayed in the middle of the screen, one at a time, and the participant had to select the box in which the pattern was originally located. If the participant made an error, the boxes were opened in sequence again to remind the participant of the locations of the patterns. Increased difficulty levels were used to test high-functioning, healthy individuals.	The test began with several coloured squares (boxes) shown on the screen. The aim of this test was that, by selecting the boxes and using a process of elimination, the participant should find one yellow ‘token’ in each of several boxes and use it to fill up an empty column on the right-hand side of the screen. Depending on the difficulty level used for this test, the number of boxes could be gradually increased until a maximum of 12 boxes were shown for the participants to search. The colour and position of the boxes used were changed from trial to trial to discourage the use of stereotyped search strategies.

## Data Availability

Data are available on request.
